# Hydroxyapatite Functionalized Calcium Carbonate Composites with Ag Nanoparticles: An Integrated Characterization Study

**DOI:** 10.3390/nano11092263

**Published:** 2021-08-31

**Authors:** Eleonora Bolli, Saulius Kaciulis, Alessio Mezzi, Valeria Ambrogi, Morena Nocchetti, Loredana Latterini, Alessandro Di Michele, Giuseppina Padeletti

**Affiliations:** 1 Institute for the Study of Nanostructured Materials, ISMN-CNR, 00015 Rome, Italy; saulius.kaciulis@cnr.it (S.K.); alessio.mezzi@cnr.it (A.M.); giuseppina.padeletti@cnr.it (G.P.); 2 Department of Industrial Engineering, University of Rome Tor Vergata, 00133 Rome, Italy; 3 Department of Pharmaceutical Science, University of Perugia, 06123 Perugia, Italy; valeria.ambrogi@unipg.it (V.A.); morena.nocchetti@unipg.it (M.N.); 4 Department of Chemistry, Biology and Biotechnology, University of Perugia, 06123 Perugia, Italy; loredana.latterini@unipg.it; 5 Department of Physics and Geology, University of Perugia, 06123 Perugia, Italy; alessandro.dimichele@unipg.it

**Keywords:** Ag nanoparticles, hydroxyapatite functionalized calcium carbonate, composite, XPS, Auger parameter

## Abstract

In the present work, composite materials very promising for biomedical and pharma-ceutical applications were investigated. They are composed of silver nanoparticles (Ag NPs) in a matrix constituted of calcium carbonate functionalized with hydroxyapatite (HA-FCC). The composites were obtained by different synthesis methods, starting from a mixture of the silver acetate with HA-FCC (using adsorption or mixing in wet conditions methods) and then treating them by exposure to visible light or calcination to promote the silver reduction; a synthetic procedure based on ultrasound-assisted reduction with NaBH_4_ or citrate was also carried out. The characterization by X-ray photoelectron spectroscopy and reflected electron energy loss spectroscopy analysis also involved the reference sample of HA-FCC matrix. Then the morphology of the Ag NPs and the crystalline structure of HA-FCC were studied by transmission electron microscopy and X-ray diffraction, respectively. To assess the effectiveness of the different methods on silver reduction, the Auger parameters α’ were calculated and compared. The use of this methodology based on the Auger parameter is neither trivial nor ordinary. We demonstrate its validity since the different values of this parameter allow to identify the oxidation state of silver and consequently to evaluate the formation yield of metallic Ag NPs in the HA-FCC matrix and the effectiveness of the different reduction methods used.

## 1. Introduction

Noble metal nanoparticles find diverse applications in many emerging scientific areas. More recently it was observed that the addition of nanoparticles as integrated element in composite materials can provide a significant improvement of their characteristics [1,2,3,4,5,6]. Indeed, the operation of these new materials relies on the peculiar properties of metal nanoparticles, such as mechanical, electronic, and optical ones [7,8,9,10,11].

Among all metal nanoparticles, Ag nanoparticles have attracted the attention of researchers for their numerous biological activities [12,13,14,15]. For their characterization, the analytical technique of X-ray photoemission spectroscopy (XPS) results to be a tool with peculiar properties capable to provide information on surface chemical composition.

By using the analytical technique of X-ray photoemission spectroscopy, it is possible to obtain not only the information on the surface chemical composition, but also to identify the oxidation state of the elements from the chemical shift of their photoemission peaks. However, in some cases it is difficult or even impossible to determine the oxidation state due to the very small value of chemical shift. This phenomenon is characteristic for some metals, also including silver [16]. The noticeable shift of photoemission peaks could be also caused by the reduced size of NPs, as it has been demonstrated for Ag and Au [17,18,19,20,21,22]. This shift, which is called size-shift, occurs when the metallic nanoparticles are of the dimension less than ≈5 nm and has been also predicted in previous theoretical works [23,24].

The material synthesized and characterized in this work consists of different composites of silver and hydroxyapatite (HA) functionalized calcium carbonate (FCC) [25,26]. These composites combine the unique properties of noble metal nanoparticles with the antibacterial action of silver [27,28] and biocompatibility of HA-FCC, consequently they could be very promising for application in biomedical and pharmaceutical fields.

The composite samples were prepared by blending HA-FCC with silver acetate (adsorption or mixing in wet conditions) followed by the physical (exposure to high-dose visible light) or chemical (calcination or high power ultrasound assisted reduction with NaBH_4_ or citrate) treatments to boost silver reduction and nanodispersion.

In the investigated composite, the diameter of Ag NPs was higher than 5 nm, hence the size-shift of XPS peaks was not expected. Therefore, the spectrum of spin-orbit doublet Ag 3d in metallic NPs should be identical to bulk Ag. However, for the identification of Ag oxidation state in NPs it is possible to acquire the main Auger signal of Ag MNN transition and to determine the Auger parameter α’ [29], which is calculated by adding the binding energy (BE) of Ag 3d_5/2_ peak to the kinetic energy (KE) of Ag MNN one.

The novelty of the present work lies on the use of X-ray photoelectron spectroscopy and reflected electron energy loss spectroscopy to investigate hybrid nanostructured materials with a quite copious and complex chemical and structural compositions, to obtain fundamental information on the Ag oxidation state in different samples. The detailed and challenging analysis to determine the Auger parameters enables to determine the sensitivity of the XPS-measurements even in hybrid samples and to establish these spectroscopies as a valuable tool to examine materials, which are relevant in many application fields.

For the comparison of results obtained for the samples prepared by the different methods, the data for all the samples were summarized in a classical Wagner plot.

## 2. Materials and Methods

### 2.1. Preparation of Ag and HA-FCC Composites

The material Omyapharm 500-OG, which was kindly provided by Omya Italia (Carrara, MS, Italy), was used as HA-FCC. Silver acetate was purchased from Alfa Aesar (Karlsruhe, Germany). Deionized water was obtained by reverse osmosis process in a MilliQ system (Millipore, Rome, Italy). Other reagents and solvents were of reagent grade and were used without further purification.

Ag and HA-FCC composites were obtained by three different methods:

1. HA-FCC (1g) was equilibrated with an almost saturated solution of silver acetate (100 mg) in deionized water (10 mL) and the composite was recovered by filtration to obtain the sample 1. Afterwards, the sample 1 was either irradiated with a Hg lamp equipped with a long pass filter to enable exposure to 430–800 nm radiation for 120 min (obtaining sample 2) or it was calcined at 300 °C to produce the sample 5;
2. A dispersion of HA-FCC (1 g) was mixed with an aqueous almost saturated solution of silver acetate, then it was heated at 37 °C until dryness (sample 3) and then irradiated as for sample 2 in order to obtain the sample 4;
3. For the synthesis of samples 6–9, a solution of reducing agent (NaBH_4_, or trisodium citrate) was dropped, under high power ultrasound irradiation for 45 min at 50% of amplitude and at 25 °C, to an aqueous dispersion of silver acetate (50 mg) and HA-FCC (1 g). The solid material was recovered by centrifugation. Two different amounts of silver acetate (50 mg and 100 mg) were used to obtain four different composites: the samples 6 and 7, when trisodium citrate was used; the samples 8 and 9, when the reducing agent was NaBH_4_). The high-power ultrasound irradiation was performed by using an Ultrasonic processor VCX750 (Sonics & Materials, Inc., Newtown, CT, USA), 20 kHz, with a diameter tip of 13 mm.

**Figure 1:**
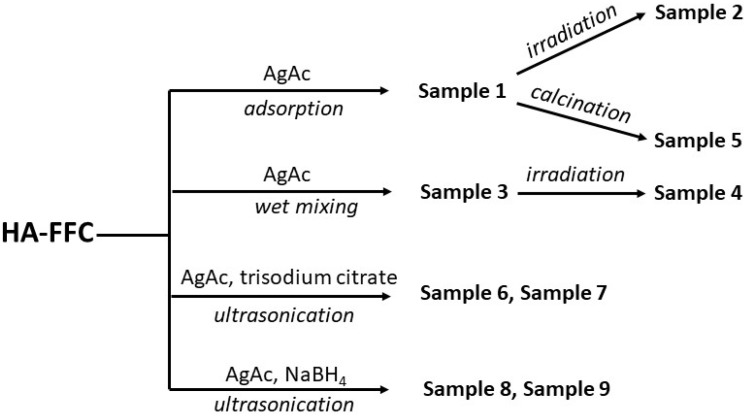
Sample preparation scheme.

The samples preparation is summarized in a scheme shown in Figure 1.

### 2.2. Characterization

X-ray powder diffraction (XRPD) patterns were taken by a Philips X’PERT PRO MPD diffractometer (Malvern Panalytical Ltd., Malvern, UK) operating at 40 kV and 40 mA, with a step size of 0.0170 2θ degree, and step scan of 20 s, using Cu Kα radiation and an X’Celerator detector (Malvern Panalytical Ltd., Malvern, UK).

Transmission electron microscopy (TEM) images were collected by a Philips 208 transmission electron microscope (FEI, Hillsboro, OR, USA). The samples were prepared by the deposition of a small drop of the aqueous dispersion of the solids on a copper grid precoated with a Formvar film and then evaporated in air at room temperature.

XPS analyses were carried out by using an electronic spectrometer Escalab MkII (VG Scientific Ltd., East Grinstead, UK). The samples, deposited on pure Au foil (99.99%) and mounted on the sample holder, were introduced into the analysis chamber, where the base pressure was maintained at ~2 × 10^−9^ mbar. All the spectra were excited with a standard unmonochromatized Al Kα source and registered by a 5-channeltron detection system. To acquire the spectral regions with high energy resolution, the analyzer was set to constant pass energy of 40 eV. To increase the signal-to-noise ratio, the Auger spectra were collected at the pass energy of 100 eV. The input slits of the analyzer have been set to large-area mode A1 × 22, providing the diameter of analyzed area of about 10 mm. The REELS experiments on the HA-FCC reference sample were carried out in an Escalab 250Xi spectrometer (Thermo Fisher Scientific Ltd., East Grinstead, UK) equipped with an in-lens electron source operating in the energy range of 100–1000 eV.

All experimental data were acquired and processed by using the Avantage v.5 software (Thermo Fisher Scientific Ltd., East Grinstead, UK). The “smart” background and mixed Lorentzian/Gaussian peak shape (30%) was used for the peak fitting routine. Scofield’s relative sensitive factors with energy compensation coefficient of KE^0.6^ were applied for the elemental quantification.

#### Preliminary Discussions

The photoemission and Auger spectra acquired for each composite contain not only the peaks of Ag NPs, but also the ones of constituent elements of HA. As it is known from the previous XPS studies on hydroxyapatite, in the spectra of O 1s are present two strong shake-up satellites at higher BE referred to the excitations from O 2s and Ca 3p levels to unoccupied P-O and Ca-O orbitals in the valence band [30,31]. It is logical to suppose that the same satellites could be also present in the spectra of Ca 2p and P 2p, moreover, these satellites of Ca 2p would overlap with the spectrum of Ag 3d. To identify all possible energy losses in photoemission spectra of composite, the measurements of reflected electron energy loss spectroscopy (REELS) were carried out for the reference sample of HA-FCC without Ag NPs. These results permitted to identify not only shake-up satellites, but also all plasmonic energy losses in HA and/or HA-FCC. Afterwards, by subtraction of Ca 2p satellites from Ag 3d spectra were obtained the photoemission signals from Ag NPs.

Before the identification of Ag oxidation state in different composite samples, the values of Auger parameter were determined for the reference compounds of Ag_3_PO_4_, Ag_2_O, and AgCH_3_COO.

## 3. Results and Discussion

### 3.1. XRD and TEM Results

**Figure 2:**
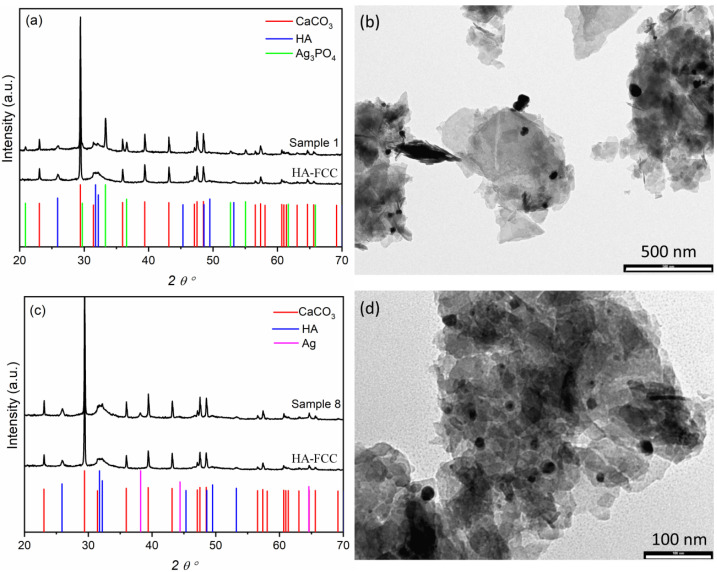
XRD pattern of HA-FCC compared with the samples 1 (**a**) and 8 (**c**). Below the spectra are reported the pattern lines of: CaCO_3_ (PDF#01-083-1762)—red lines, HA (PDF#01-086-0740)—blue lines, Ag_3_PO_4_ (PDF#01-084-0192)—green lines, Ag (PDF#87-597)—magenta lines. TEM images of the samples 1 (**b**) and 8 (**d**).

Figure 2a shows the XRD pattern of HA-FCC compared with that of the sample 1. The CaCO_3_ and HA phases in the HA-FCC composite are clearly detected, whereas the treatment with silver acetate induces the formation of a new phase identified as Ag_3_PO_4_ by comparison with the reference spectra of cubic silver phosphate (PDF#01-084-0192). The diameter of Ag_3_PO_4_ particles calculated by the Debye-Scherrer equation was of 79 nm, which is in good agreement with average dimension of 70 ± 36 nm determined for 50 particles of Ag_3_PO_4_ visible in the TEM image reported in Figure 2b. Conversely, the treatment of HA-FCC with silver acetate in the presence of NaBH_4_ gives preferentially Ag nanoparticles as highlighted by the reflections of (*111*) and (*200*) crystalline planes of the cubic metallic Ag (PDF#87-597) in the XRD pattern of the sample 8 (Figure 2c). TEM images in Figure 2d show dark nanoparticles with a diameter of about 20 nm ascribable to Ag.

### 3.2. XPS Results

#### 3.2.1. HA-FCC Reference Sample

**Figure 3:**
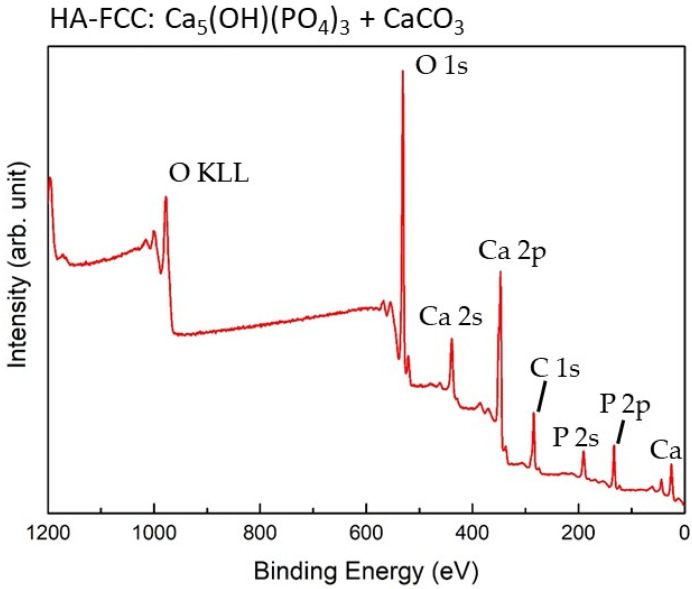
Survey spectrum of HA-FCC reference sample. The main peaks of P, Ca, C and O are indicated.

The typical survey spectrum of HA-FCC reference sample is shown in Figure 3 and its chemical composition is reported in Table 1. From the quantitative analysis of the revealed elements, it is possible to evaluate the atomic ratios of Ca/P and O/Ca. Obtained values of Ca 2p/P 2p ~1.5 and O 1s/Ca 2p ~2.5 are almost in agreement with stoichiometric compound, i.e., Ca_5_(OH)(PO_4_)_3_ + CaCO_3_. Besides, it should be noted the absence of any impurities except of 15.4 at% of adventitious carbon, corresponding to the component C1s-A at a binding energy (BE) of 285.0 eV listed in Table 1.

**Figure 4:**
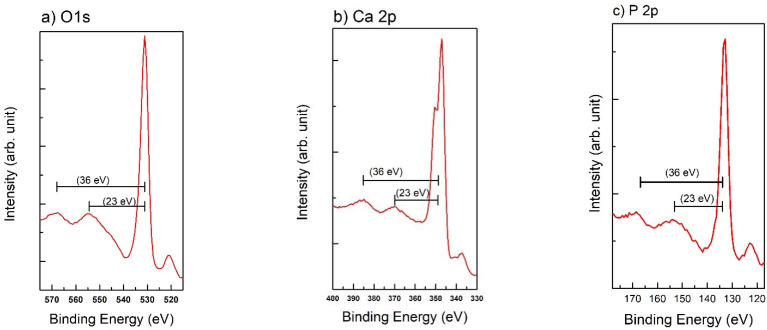
XPS spectra of O 1s (**a**), Ca 2p (**b**), and P 2p (**c**) regions. Besides the main photoemission peaks, in all the spectra are clearly visible the shake-up satellites.

Two strong satellites, shifted to higher BE by 23 ± 1 eV and 36 ± 1 eV, are well visible in the O 1s region. The same satellites are present also in the photoelectron regions of Ca 2p and P 2p reported in Figure 4.

**Figure 5:**
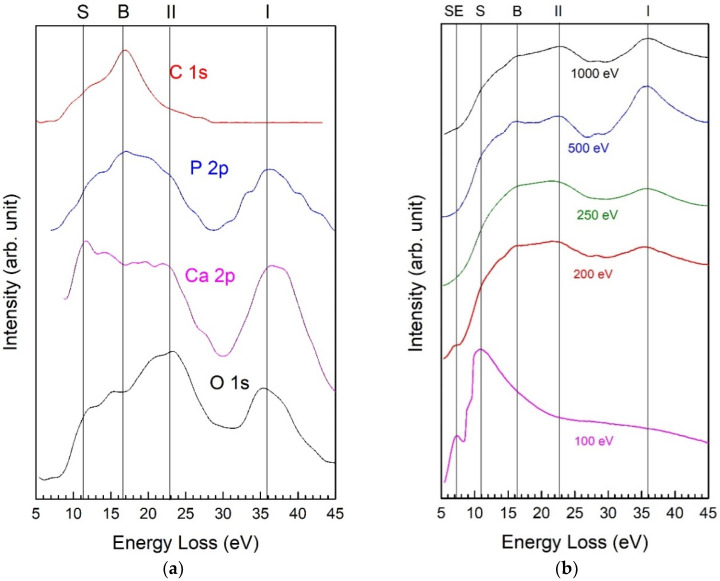
Electron energy losses in XPS spectra (**a**) and REELS spectra recorded in a primary energy range of 100–1000 eV (**b**) for the HA-FCC reference sample.

Furthermore, as shown in Figure 5a, some other energy losses are repeating in the photoemission regions of the main constituents of HA-FCC. In order to clarify the origin of these loss peaks in hydroxyapatite functionalized calcium carbonate, the REELS analyses were performed on the reference sample. By decreasing the primary electron energy from 1000 to 100 eV, the surface sensitivity of REELS measurements was gradually increased. The results, obtained for all the energies of primary beam, are shown in the Figure 5b.

Two strong peaks at 36 and 23 eV (marked as I and II), actually are the same shake-up satellites referred to the excitations from O 2s and Ca 3p to unoccupied orbitals of (P-O) and (Ca-O) in the valence band, respectively, observed also in photoemission spectra [30,31]. Sometimes these peaks are confused with collective electronic excitations, i.e., plasmonic losses, as it has been referred to in [32,33]. Instead from comparison with REELS spectra, it is clear that the other peaks at about 11.0 and 16.5 eV from the elastic peak, are attributed to the bulk (B) and surface (S) plasmonic losses in the crystalline HA-FCC structure [34]. In fact, the plasmon excitations B and S are visible even in C 1s spectral region, where are absent the losses I and II of 36 and 23 eV, because in the excitation of shake-up satellites are involved only O, Ca, and P electronic orbitals.

When the loss spectra are excited by low energy electrons of 200 and 100 eV, their energy becomes too low for the excitation of shake-up processes and bulk plasmons, therefore, these loss peaks are disappearing from the spectra. At 100 eV, the principal energy losses are caused by the surface plasmons S at 11.0 eV and surface excitons SE at 7.3 eV [34].

#### 3.2.2. Reference Samples of Ag

Three silver compounds were analyzed to obtain the reference spectra: AgCH_3_COO, which is the precursor used to produce the nanoparticles in composite, Ag_3_PO_4_ and the silver oxide Ag_2_O. In the first two columns of the Table 2 are reported the BE values of the main doublet peak Ag 3d_5/2_ and the value of kinetic energy (KE) of Auger transition Ag M_4_N_5_N_5_. The BE scale was calibrated by setting the C 1s peak of adventitious carbon at 285.0 eV. Consequently, by rescaling the spectra to eliminate possible sample charging, the Ag 3d_5/2_ peaks of phosphate Ag_3_PO_4_ and acetate AgCH_3_COO were located at BE = 368.4 eV, whereas for Ag_2_O it was at BE = 367.9 eV. Since the chemical shift in silver photoemission peaks is too small for accurate identification of Ag chemical state [20], it was necessary to also acquire the Auger spectra, where the chemical shift in different silver compounds is higher, even of some eV. Then it is possible to calculate the Auger parameter α’ by adding the BE of Ag 3d_5/2_ peak to the KE of corresponding Auger transition Ag M_4_N_5_N_5_:

α’ = BE (Ag 3d_5/2_) + KE (Ag M_4_N_5_N_5_)

In the case of low intensity Auger signals, as it often happens in the composites with Ag nanoparticles, it is better to use the higher intensity peak of Ag M_5_N_5_N_5_ and to add 6.0 eV, corresponding to the difference in KE between two Auger peaks [35]. Then, from the calculated values of Auger parameter it is possible to determine the oxidation state of silver.

For the reference sample of Ag_2_O, the Auger parameter was equal to 724.2 eV, which perfectly corresponds to the oxidation state +1 in this oxide [35]. The values of Auger parameter determined for the reference samples of Ag_3_PO_4_ and AgCH_3_COO were slightly lower, in the range of α’ = 722.5–722.7 eV, as it has been reported for other Ag salts with Ag^+1^ [29,35,36,37]. All these results for three reference samples are summarized in Table 2.

#### 3.2.3. Composite Samples with Ag NPs

After the preliminary analyses done on the reference samples, the investigations were continued on the composite materials of HA-FCC with silver, prepared using AgCH_3_CO_2_ precursor. The results obtained for different HA-FCC + AgCH_3_COO samples are reported in this chapter. Different samples were prepared by using three methods specifically, (1) adsorption followed by irradiation or calcination, (2) wet mixing followed by irradiation, (3) ultrasound assisted reduction with citrate or NaBH_4_. Irradiation and calcination should act as silver reduction method [38,39,40,41] stimulating the formation of Ag nanomaterials homogeneously dispersed in HA-FCC. As it will be described below, different results have been obtained for each method.

**Figure 6:**
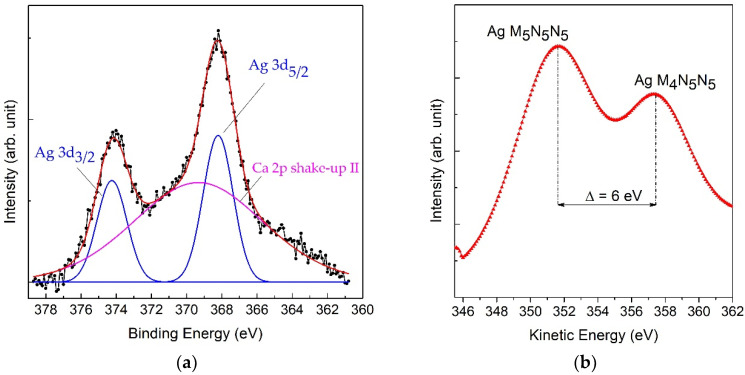
XPS regions of Ag 3d photoemission (**a**) and Ag MNN Auger transition (**b**) for the sample reduced with NaBH_4_ (Sample 8 reported in Table 3).

For all the composite samples before and after the treatments, the main photoemission peak of Ag 3d falls in the same spectral region with the first shake-up satellite (marked II in Figure 5) of Ca 2p line. Therefore, it was necessary to include this satellite in the peak fitting of Ag 3d spectra, as it is shown in Figure 6. For each sample, all the main peaks of HA-FCC including their shake-up satellites have been also acquired: O 1s at BE = 531.5 eV, Ca 2p at BE = 347.7 eV, and P 2p at BE = 133.7 eV. The position of these reference peaks was used to check the calibration of BE scale. In addition to shake-up satellites, also the plasmonic losses were observed in all the spectra, indicating that the crystalline structure of HA-FCC has not been altered in the composite.

The study of the formation of Ag NPs, starting from the mixture with precursor and during the process of reduction, was continued by following the variation of silver Auger parameter. Therefore, all obtained results were compared with the reference samples and with references reported in literature [35,36,37]. The results obtained for all the samples of HA-FCC + AgCH_3_COO before and after all the treatments (irradiation, calcination, chemical reduction under sonication) are summarized in Table 3.

The first method used to mix the AgCH_3_COO with HA-FCC, so-called adsorption (sample 1 in Table 3), resulted in Auger parameter α’ = 722.5 eV. It is not possible to distinguish, if this value is attributable to an oxidation state +1 in the silver acetate itself or to the formation of possible Ag phosphate bonds with phosphorus in HA-FCC. As regards the second method of mixing in wet conditions, corresponding to sample 3 in Table 3, it was characterized by the value of α’ = 722.7 eV. In this case, the value corresponds to the oxidation state +1. The value of Auger parameter, obtained for the compound mixed by the absorption method and then subjected to irradiation, was of 723.9 eV. This difference of α’ before and after irradiation indicates a change of the Ag oxidation state. Therefore, a partial silver reduction was observed, but it was not sufficient to produce metallic nanoparticles. Calcination also did not produce a sufficient reduction: in this case the Auger parameter was α’ = 723.6 eV, which is also attributable to silver oxide Ag_2_O. Better results were obtained after the sonication/chemical reduction treatment: α’ = 724.9 and 725.7 eV for the samples 6 and 7, respectively, whereas the addition of reducing agent NaBH_4_ resulted in α’ = 725.7 and 725.4 eV for the samples 8 and 9, respectively. In this case was identified the state of metallic silver, indicating the formation of Ag NPs.

**Figure 7:**
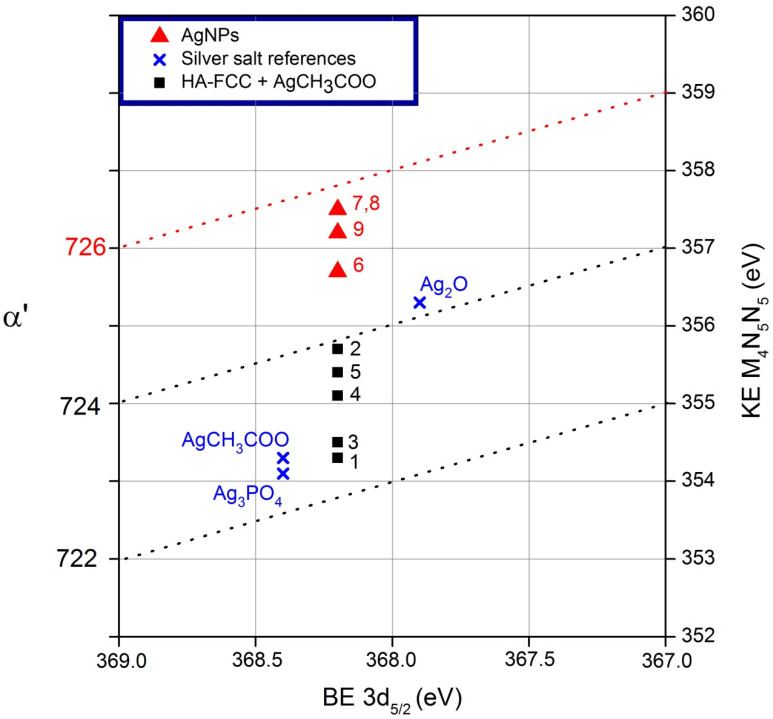
Wagner plot for the experimental results of Auger parameter determined for all the samples. Sample numbers are also indicated in the plot. Dashed lines for eye guidance—constant values of α’. The values for Ag NPs lie near to the literature data for metallic Ag (red dotted line).

The results obtained for all the samples, including those of the three reference compounds, were summarized in the classic Wagner plot presented in Figure 7.

The determination of Ag oxidation state by using the Auger parameter for identification of metallic Ag and consequently of the NPs formation is fast and reliable. This method has already been used successfully for other types of compounds with Ag nanoparticles in solution [18,19].

## 4. Conclusions

The composites of Ag nanoparticles dispersed in hydroxyapatite HA-FCC were synthesized by using different methods and characterized by XRD, TEM, REELS, and XPS techniques.

The REELS analyses of the HA-FCC reference sample have identified the nature of two strong satellites, shifted to higher BE by 23 ± 1 eV and 36 ± 1 eV from the main XPS peaks of O 1s, Ca 2p, and P 2p. All these losses were attributed to the shake-up satellites referred to the excitations from O 2s and Ca 3p core levels to unoccupied orbitals of (P-O) and (Ca-O) in the valence band and not to plasmonic collective excitations as it has been often confused. Nevertheless, also the plasmonic losses were present in the REELS spectra at about 16.5 eV (bulk plasmon) and 11 eV (surface plasmon) with respect to the elastic peak.

The use of the Auger parameters in determining the effectiveness of the different silver reduction methods, proved to be successful. In fact, from the calculated Auger parameter α′ for all the investigated samples, it was possible to evaluate the outcome of the different silver reduction methods in composite. Some of these methods, such as irradiation and calcination, were not entirely effective, producing only a partial reduction of silver in NPs. Instead, the other methods such as the combination of sonication with the addition of citrate or NaBH_4_, led to a complete reduction of silver to metallic state, indicating the formation of Ag NPs with a diameter of about 20 nm.

## Figures and Tables

**Table 1:** XPS quantitative chemical composition of HA-FCC reference sample.

Peak	BE, eV (±0.1 eV)	Atomic %	Bonds
C1s-A	285.0	15.5	Adventitious carbon
C1s-B	287.0	2.2	C=O
C1s-C	289.9	2.4	−C=O
Ca2p_3/2_	347.7	19.3	Ca^2+^
O1s-A	531.6	41.5	Phosphate, C−O, OH^−^
O1s-B	533.9	5.9	C=O, H_2_O
P2p_3/2_	133.3	13.3	Phosphate

**Table 2:** Values of Ag 3d_5/2_ (in BE), Ag M_4_N_5_N_5_ (in KE), Auger parameter and corresponding silver oxidation state for the reference samples of Ag compounds.

Compound	Ag 3d_5/2_ BE, eV (±0.1 eV)	Ag M_4_N_5_N_5_ KE, eV (±0.1 eV)	α’, eV (±0.2 eV)	Ag Oxidation State
Ag_3_PO_4_	368.4	354.1	722.5	+1
Ag_2_O	367.9	356.3	724.2	+1
AgCH_3_COO	368.4	354.3	722.7	+1

**Table 3:** Values of Ag 3d_5/2_ BE, Ag M_4_N_5_N_5_ KE, Auger parameter α’ and Ag oxidation state for all the composite samples before and after reduction treatments.

Composite Samples with Ag	Ag Reduction Treatment	Ag 3d_5/2_ BE, eV (±0.1 eV)	Ag M_4_N_5_N_5_ KE, eV (±0.1 eV)	α’, eV (±0.2 eV)	Ag ox. State
Sample 1	Adsorption	368.2	354.3	722.5	+1
Sample 2	Adsorption and irradiation	368.2	355.7	723.9	+1
Sample 3	Wet mixing	368.2	354.5	722.7	+1
Sample 4	Wet mixing and irradiation	368.2	355.1	723.3	+1
Sample 5	Adsorption and Calcination	368.2	355.4	723.6	+1
Sample 6	Sonication/citrate	368.2	356.7	724.9	+1 metallic
Sample 7	Sonication/citrate	368.2	357.5	725.7	metallic
Sample 8	Sonication/NaBH_4_	368.2	357.5	725.7	metallic
Sample 9	Sonication/NaBH_4_	368.2	357.2	725.4	+1/metallic
